# Miscellaneous

**Published:** 1859-03

**Authors:** 


### MISCELLANEOUS.

### THE WHITNEY CASE.

We copy the following from the March number of the *Virginia Medical Journal*, in regard to the celebrated Whitney case:

"The medical profession of New York, and indeed the public generally, have been in a state of very considerable excitement for the past few weeks, caused by the sudden death of Mr. S. S. Whitney, the only son of a well known millionaire, under peculiar circumstances. The newspaper press of that city being in want, just then, of anything likely to interest and excite their readers, seized upon the opportunity afforded them of making some good paragraphs, and inasmuch as the Whitneys had money—the one thing needful in Gotham—and the causes of death admitting of very considerable difference of opinion, the whole affair has gained a notoriety, reflecting but little credit upon any of the parties concerned. But for the poor man's money, he would have gone to a quiet and unnoticed grave; the doctors would have never fought over his diseased carcass, and the public would have been ignorant of his dissipations and follies. But a patient worth a *million* cannot be parted with so easily; and thus the jealousies and squabbles of the profession have been laid before the public.

"Mr. Whitney, a man of irregular habits, weakly constitution, and capricious temper, having broken himself down, as fast young men do frequently, becomes a confirmed invalid; and as there was phthisis in the family, he presently finds his way to Dr. Horace Green.

"This gentleman has been for years levying a tribute upon that large class of sufferers, who are marching along the slow but certain road, which begins with consumption and ends with the grave. He is the gentleman who revived the old practice, which had never received much attention, of attempting to cure affections of the throat by the local cautery; and as the idea has much merit in it, a reputation was soon made with a certain class of patients. Clergymen, especially, said to have lost their voices from over-work, flocked to him in numbers, with purses filled by fond congregations; and as these patients generally had *follicular pharyngitis*, the result of good dinners, cigars and lazy habits, Dr. Green had an easy task before him. A professor in a medical school speaking an hour every day, or a lawyer talking all the time, rarely complains of that affection, called *clergyman's sore throat;* and yet they use their organs of voice much more constantly. The disease is often a secondary affection, beginning with the stomach—to be cured by traveling, change of diet, and the local treatment.

"Dr. Green, however, made a good thing of this practice, and deserves credit, in our opinion, for calling attention to a method of treatment not without value. In his next movement in the same direction, there was also much to praise. He proposed to introduce caustic solutions through the larynx into the trachea, in cases of croup and laryngitis, both acute and chronic; and while some may doubt whether he really does pass his *probang* between the vocal chords as often as is claimed for him, yet certainly the fluid does pass, and is productive of benefit.

"The next step, however, was still more daring. Dr. Green proposes to *catheterize the trachea,* and inject the solution of caustic into the cavities of the lungs; and this startling proposition has drawn to his consultation rooms many patients, who, having death before their eyes, desperately resort to any one who promises a cure.

"Mr. Whitney seems to have been one of these persons. He goes to Dr. Green, knowing what his treatment was; submits to all his applications; and one day after his visit, he goes home, declares that Dr. Green has killed him; sends for Drs. Mott and Beales; and after a week of suffering with dyspnœa, accompanied with emphysema of the neck, face and thorax, he at last succumbs. Dr. Green is not present during this time, having been discharged with ignominy, and the two gentlemen who succeeded him did not insist on his presence. Even at the post mortem he was not permitted to have a hearing; and the whole city of New York conclude that an abscess formed in the pharynx was made by his caustic, and a cavity in the lung, which had been opened through the pleuræ into the cellular tissue, and caused the emphysema, was a puncture with the point of the instrument.

"Dr. Green and his friends rally to the rescue, and the affair is brought before the Academy of Medicine. Then comes a grand set-to. Every body says something; gives an opinion; proposes a theory. Mr. Whitney is killed in a half dozen ways. Charges are made and denied; and at last, exhausted of their venom, all parties agree to pass a resolution unanimously declaring that Whitney died because his time had come.

"What a lesson can be drawn from this whole controversy, showing the absolute necessity for the most stringent and inviolable rules to govern the profession in their intercourse with each other.

"Dr. Green was either a charlatan or not. If he deceived and pillaged the public, and risked wantonly the lives of his patients, he ought to have been cut by the profession, turned out of the academy, and not regarded as a physician.

"But if Dr. Green was held in full fellowship, he had a right to be defended by his brethren. No one ought to have taken charge of Whitney without his knowledge; and he certainly had the indefeasible right, in common justice, to have witnessed the autopsy.

"Why should Whitney or his friends complain of a practice to which he knowingly resorted? Dr. Green makes no secret of his procedures; and under the circumstances it was entirely wrong in any physician to take charge of the case—still worse, to express opinions derogatory to the character of an absent brother. If any other course is allowed, there will be no end to Whitney cases. Every death will be brought up against us, and post-mortems, controversies and suits for malpractice will fill up our time. If a man is a quack, and a credulous public choose to patronize him, let them die on his hands. If he is honest, candid, and fairly endeavors to administer the benefit of the art to the sick, he can do no more, and his brethren should stand by him, and shield him from censure.

Draw the lines which divide the true physician from the unworthy pretender, as strongly as possible. Require every member to submit to the rules and discipline necessary for self-protection, and sternly carry these principles into effect. It is the only way to get rid of the dishonest practitioner. Any other course leads to disgrace and confusion, and our honorable calling is made the laughing stock of the world.

---

At the last annual meeting of the State Medical Society, the undersigned was appointed Chairman of the Committee of Surgery, whose duty it is to report at its next annual meeting, to be held at Decatur, June 7th, 1859. "On all the important improvements in the management of surgical diseases effected in Illinois during the year."

Being desirous of making the report as full and complete as possible, he takes this opportunity of requesting the profession of the State to aid him in the same, without which the report cannot be anything but imperfect.

Let every physician in the State, therefore, who has anything new or interesting, either in the science or art of surgery (and every one must have something), enclose the same, carefully prepared, to the undersigned, on or before the 1st of May next.

Especially statistics and reports of operations, viz.: amputation, lithotomy, etc., will be gladly received.

All matters received will be duly accredited to the author.

EDWIN POWELL.

The undersigned was appointed Chairman of the Committee on Obstetrics and Diseases of Women and Children, at the Rockford meeting of the Illinois State Medical Society, in June last. Being desirous of eliciting the aid of the profession in the State in making a report on these important subjects, he takes this method of requesting physicians who may have any original information relative to them, or interesting and rare cases, to send it to him by the 1st of May next. Any matter thus furnished will be duly accredited to the author, and thankfully received. Communications of this kind may also be addressed to Drs. D. W. Young, of Aurora, Ill., and H. Noble, Independence, McLean Co., Ill., his associates on the committee.

W. H. BYFORD.

Chicago, Feb. 19th, 1859.

### AMERICAN MEDICAL ASSOCIATION.

The twelfth meeting of this association will be held in Louisville, Ky., on Tuesday, May 3d, 1859. The secretaries of all societies, and other bodies entitled to representation in the association, are requested to forward to the secretary, S. M. Bemiss, at Louisville, correct lists of their delegations as soon as they may be appointed.

The convention of teachers, invoked by a resolution of the National Association, for the purpose of a general conference upon the best means of elevating the standard of medical education in this country, will meet on Monday, May 2d.

Medical journals throughout the United States, are requested to insert the above. S. M. BEMISS,
Secretary Am. Med. Association.

### INTERMURAL INTERMENTS,

In the city of London, have, under the act of Parliament, regulating the subject, almost entirely ceased. Most of the graveyards are now closed, and the remainder will, by law, soon discontinue to be used for interments.

### DEATH OF DR. MUTTER.

The late Philadelphia journals announce the death of Thomas D. Mutter, late Professor of Surgery in the Jefferson Medical College. Dr. Mutter, it is well known, has been in ill health for some years. A few months ago, he returned from a European tour, which he had taken for the benefit of his health, and has been spending a portion of the winter in the South, on the same account. He died at Charleston, S. C. He was about sixty years of age. The news of his death wiil cast a gloom over a very large circle of professional friends in all parts of our own and in other countries who have sat under his teachings.

### AMPUTATION AT THE HIP JOINT.

This operation was performed by Professor Brainard, Feb. 24, 1859, for necrosis of the femur, extending from the knee joint to the cervix. The patient died eighteen hours after the operation.

Professor Paul Eve, in his report to the American Medical Association for 1851, notices the following cases of this amputation, which had then been reported in the United States:

1. By Dr. Mathew Beashea, of Beardstown, Ky., successful.
2. By Dr. Mott, for necrosis of femur.

3. By Professor Brainard. Patient recovered, but died from return of the disease, which was enchondroma.
4. By Professor Wm. Van Buren. Successful.
5. By Professor May. Successful.
6. Drs. Richards and Clagett, of Maryland. Successful.

